# Antibiotic-Free Strategies for Managing Antimicrobial-Resistant Infections

**DOI:** 10.3390/pharmaceutics18091136

**Published:** 2026-09-10

**Authors:** Payal Ganguly, Elena A. Jones, Peter V. Giannoudis, Giuseppe Tronci

**Affiliations:** 1 Leeds Biomedical Research Centre, Leeds Teaching Hospitals NHS Trust, Leeds LS7 4SA, UK; 2 Leeds Institute of Rheumatic and Musculoskeletal Medicine, Faculty of Medicine and Health, University of Leeds, Leeds LS9 7TF, UK; 3 Academic Department of Trauma and Orthopaedic Surgery, School of Medicine, University of Leeds, Leeds LS2 9JT, UK; 4 School of Dentistry, St. James’s University Hospital, University of Leeds, Leeds LS9 7TF, UK; 5 Clothworkers’ Centre for Textile Materials Innovation for Healthcare (CCTMIH), School of Design, University of Leeds, Leeds LS2 9JT, UK

**Keywords:** antimicrobial resistance (AMR), antibiotic-free strategies, natural antimicrobials, nanoparticles (NPs), polymers, metal–organic frameworks (MOFs), infection management

## Abstract

Antimicrobial resistance (AMR) presents a global health challenge that is projected to cause nearly 2 million deaths annually by 2050. With a growing ageing population and the rise of antibiotic-resistant strains globally, developing effective alternatives to combat infections while reducing AMR risks is critical. Over recent decades, antibiotic-free strategies have emerged as promising approaches, offering advantages over traditional antibiotics and reducing the likelihood of resistance. This review highlights antibiotic-free antimicrobial strategies for applications in chronic wound care, hard tissue repair, personal protective equipment, as well as hospital hygiene and infection control. We begin by outlining the broader AMR problem and then examine natural strategies—including the use of agents such as manuka honey—followed by synthetic approaches involving nanomaterials and metal–organic frameworks (MOFs), as well as biological and bioinspired antimicrobial strategies, involving antimicrobial peptides, antibodies, bacteriophages, extracellular vesicles, and macrocycles. Finally, we discuss innovations in artificial intelligence (AI)-assisted antimicrobial development and stimulus-responsive materials, before introducing a conceptual design framework for antibiotic-free antimicrobial materials. This review provides a comprehensive assessment of emerging antibiotic-free strategies to guide the development of next-generation antimicrobial solutions that reduce reliance on antibiotics and mitigate AMR in clinical and healthcare settings.

## 1. Introduction

Antimicrobials (antibiotics, antivirals, antifungals, and anti-parasitics) are drugs employed for treating infections and infectious diseases in humans, plants and animals. As microorganisms evolve with enhanced resistance mechanisms and, in some cases, increased virulence, antimicrobial resistance (AMR) has emerged as a major global health threat. This problem is compounded by the lengthy, costly and high-risk process of developing new antibiotics and the limited effectiveness of existing alternatives. Consequently, estimates suggest that AMR could cause nearly 2 million deaths annually by 2050 [1].

According to the World Health Organisation (WHO), AMR occurs when bacteria, viruses, fungi and parasites no longer respond to antimicrobial treatments, thereby increasing the risk of severe diseases and mortality [2]. In 2019 alone, AMR caused 1.27 million deaths and contributed to nearly 5 million fatalities worldwide. This socioeconomic burden is disproportionately higher in low- and middle-income countries (LMICs), where resistance rates are reported to be 3–4 times greater than those in high-income regions. Globally, AMR generates up to $639 billion in hospital-related expenditures, with additional productivity losses estimated at $194 billion [3]. These economic and clinical burdens are expected to rise due to the increasing prevalence of multidrug-resistant pathogens and their detrimental impact on established approaches to infection prevention and treatment, including surgery, chemotherapy, and wound care. There is therefore a critical need for innovation and development of alternative antimicrobial strategies.

Traditional antibiotics are becoming increasingly ineffective as pathogenic microbes continue to develop resistance. Chronic wounds and implant-associated infections illustrate this challenge, where persistent microbial colonisation and impaired healing require sustained antimicrobial action [4]. However, antibiotics often fail to penetrate biofilms and may disrupt host microbiota, leading to further complications and delayed recovery. Moreover, the antibiotic development pipeline has largely stagnated due to economic and regulatory barriers to translation, with few truly novel classes introduced in recent decades [5,6,7]. This intensifies the urgency and creates an opportunity for antibiotic-free strategies that provide targeted, biocompatible, and AMR-compliant alternatives for infection management [8,9]. These approaches should ideally be able to reduce infection burden, preserve microbiome integrity, and support tissue repair. These features are particularly critical in chronic wound management and hard tissue repair, where microbial balance and immune modulation play a major role.

Several innovative antibiotic-free strategies have emerged over the past 10–15 years. Bioinspired molecules, such as antimicrobial peptides (AMPs), as well as natural extracts, including flavonoids and essential oils, exhibit broad-spectrum activity and immunomodulatory effects. These, together with recent innovation in AI-driven design, make AMPs promising for topical wound and hard tissue repair applications [10,11,12]. In parallel, extracellular vesicles (EVs) derived from mesenchymal stem/stromal cells (MSCs) or immune cells, have recently demonstrated regenerative, immunomodulatory, and antimicrobial functionalities suitable for infection management and tissue repair [13,14]. Nanoparticles, particularly silver (Ag) [15,16], zinc oxide (ZnO) [17,18], and carbon-based nanomaterials [19,20], exert antimicrobial effects through membrane disruption and oxidative stress [21,22], effectively targeting both planktonic cells and biofilms.

To achieve localised treatments and minimise systemic side effects, metal–organic frameworks (MOFs) offer tunable porosity and environment-triggered biodegradability, facilitating the controlled delivery of antimicrobial agents [23,24]. Functional polymers, including smart and stimulus-responsive materials, further enable controlled drug release, which is critical for achieving therapeutic efficacy while minimising cytotoxicity [25,26,27]. Such systems can deliver antimicrobials on demand in response to environmental shifts, including changes in pH and temperature, or following external stimuli, such as mechanical stimulation, improving drug retention, reducing off-target effects, and enhancing patient compliance [28,29,30]. Processing these materials into textile fibres further expands their clinical applicability, particularly for wound dressings and regenerative scaffolds, owing to the conformability, mechanical robustness and large surface area intrinsic to textile-based platforms [31,32,33,34].

Collectively, these emerging strategies represent a paradigm shift beyond conventional antibiotic therapy, incorporating approaches that combine pathogen control with microenvironment modulation, tissue repair, and adaptive antimicrobial functionality. Their integration into clinical practice has the potential to transform wound care, surgical applications, and infection prevention, mitigating AMR while improving patient outcomes.

## 2. Current Antibiotic-Free Approaches

Nature offers a diverse array of antimicrobial agents that have long been exploited for infection management, especially in the pre-antibiotic era. Several of these naturally derived antimicrobials are still used today, including transition metals such as silver and copper, as well as biological mixtures such as honey and its derivatives.

### 2.1. Naturally Existing Materials

Silver is a transition metal with well-established antimicrobial activity, whose medicinal use, e.g., blood purification, dates back to around 980 AD [35]. Today, silver-loaded antimicrobial products are routinely used in clinics, e.g., in wound dressings. Their antimicrobial action relies on the oligodynamic effect [36], whereby contact with moisture leads to ion release and damage of DNA and RNA, disrupts cell membranes, and inhibits enzymatic function. Whilst silver is effective against a broad range of planktonic bacteria and biofilms, concerns related to environmental impact and human toxicity challenge its long-term applicability. To address this, combinatorial antimicrobial testing of silver (Ag), zinc (Zn), copper (Cu), cadmium (Cd), and nickel (Ni) revealed that Ag supplemented with Cu/Ni and Zn increased prokaryotic cell permeability at sub-inhibitory concentrations, enabling an antimicrobial effect with reduced toxicity [37]. However, considerations must be given when applying these combinations, particularly those containing Cd and Ni, owing to the established cytotoxicity and genotoxicity risks associated with these materials [38,39].

Gold (Au) represents another antimicrobial transition metal of interest. Ionic gold species (Au^1+^, Au^2+^, Au^3+^) and gold nanoparticles (NPs) have demonstrated antimicrobial activity, albeit generally lower than that of conventional antibiotics. Proposed mechanisms include disruption of the bacterial membrane through ATPase binding, ATP depletion, and inhibition of tRNA binding to ribosomes [40,41]. Dasari and Yu demonstrated antimicrobial activity of gold ions against multiple bacterial strains, particularly *Pseudomonas aeruginosa* [42]. Subsequent studies confirmed strain-dependent antimicrobial effects of ionic gold (via zone-of-inhibition assays and minimum inhibitory and bactericidal concentrations) and highlighted the importance of assessing toxicity.

Copper (Cu) is another transition metal essential for normal biological function, playing key roles in protein structure, enzymatic activity and cellular regulation [43]. Its antimicrobial properties have been exploited since ancient times and are mediated by Cu ions (Cu^2+^ and Cu^3+^) that damage bacterial cell walls and membranes, promote reactive oxygen species (ROS) generation, and cause irreversible metabolic disruption in bacteria and fungi [44]. The Cu^1+^/Cu^2+^ redox cycle, Cu^3+^ ions, and associated superoxide formation play a principal role in the contact-killing mechanism [45] and rapid bacterial inactivation [46]. Thus, the use of Cu NPs and Cu-based complexes has been explored to target resistant microbial strains [47].

Beyond these widely studied metals, nickel (Ni), iron (Fe), cobalt (Co), manganese (Mn), vanadium (V), chromium (Cr) and zinc (Zn) have also demonstrated antimicrobial activity, particularly when formulated as nanoparticles (cfr. Section 3.2). In parallel, increasing attention has been directed towards metal complexes, where chirality has emerged as a valuable design parameter. Chiral metal complexes consist of non-superimposable mirror-image enantiomers and can exhibit enhanced selectivity and differential interactions with biological targets, offering new opportunities for antimicrobial development [48].

**Honey.** Honey is composed primarily of sugars and water, with minor quantities of proteins, minerals, antioxidants, and phytochemicals. The composition of honey varies according to botanical source, environmental conditions, and processing methods [49,50]. Among different honey types, Manuka honey has attracted particular attention because of its enhanced antimicrobial potency against both Gram-positive and Gram-negative bacteria. Several studies have demonstrated superior activity against *Staphylococcus aureus* and *Enterococcus faecalis*, with efficacy often linked to its high methylglyoxal (MGO) content and associated phenolic compounds [51,52]. In addition, Manuka honey has been shown to enhance the activity of selected antibiotics and, in some cases, restore susceptibility in resistant bacterial strains. The antimicrobial activity of honey is multifactorial and arises from osmotic effects, acidity, hydrogen peroxide generation, and bioactive phytochemicals [53,54]. Phenolic acids contribute through distinct mechanisms, including oxidative stress induction, membrane disruption, and leakage of intracellular contents [50,55]. The coexistence of multiple antimicrobial pathways is believed to contribute to the low propensity for resistance development associated with honey. These properties have supported its continued use in wound management and infection control. However, clinical translation remains constrained by batch-to-batch variability, potential contamination of unsterilised products, rapid diffusion from the application site, and uncertainty regarding optimal dosing and treatment regimens. Consequently, advanced formulations and controlled-release delivery systems are being explored to improve reproducibility and therapeutic efficacy [56]. Further research is required to achieve polymeric carriers with controlled release kinetics, while systematic testing of product purity, sterility, and antimicrobial activity is essential to develop standardised clinical guidelines for dosing, formulation, and application frequency.

**Phytochemicals.** Plant-derived phytochemicals represent another major class of naturally occurring antimicrobial compounds. These secondary metabolites encompass a wide range of chemical families, including flavonoids, tannins, terpenoids, alkaloids, saponins, polyphenols, glucosinolates, and sulfinates, many of which exhibit antibacterial activity against clinically relevant pathogens [57]. Among these, flavonoids are the most extensively studied and exert antimicrobial action through inhibition of bacterial growth, interference with enzymatic function, and disruption of cell wall integrity [58,59]. Tannins and terpenoids have also demonstrated activity against multidrug-resistant (MDR) bacteria and have shown potential in applications ranging from wound care to oral health [60].

Polyphenols are particularly notable due to their broad biological activity. Curcumin, the principal bioactive component of turmeric, has received considerable attention owing to its anti-inflammatory, antioxidant, and antimicrobial properties [61,62,63]. Curcumin exhibits activity against a range of pathogens, including methicillin-resistant *S. aureus* (MRSA), *Enterococcus faecalis*, and *Streptococcus pyogenes*, although susceptibility varies considerably among species and strains. Beyond healthcare applications, phytochemicals have also demonstrated utility in food preservation and infection prevention [64].

Despite their promising antimicrobial properties, many phytochemicals suffer from poor aqueous solubility, limited stability, and low bioavailability, restricting their clinical utility. Recent advances in materials science have addressed these limitations through nanoformulation strategies that enhance stability, uptake, and antimicrobial performance. Such approaches highlight the growing convergence between naturally derived antimicrobials and advanced biomaterial-based delivery systems, which are discussed in Section 2.3.

### 2.2. Metal-Based Nanoparticles

**Figure 1:**
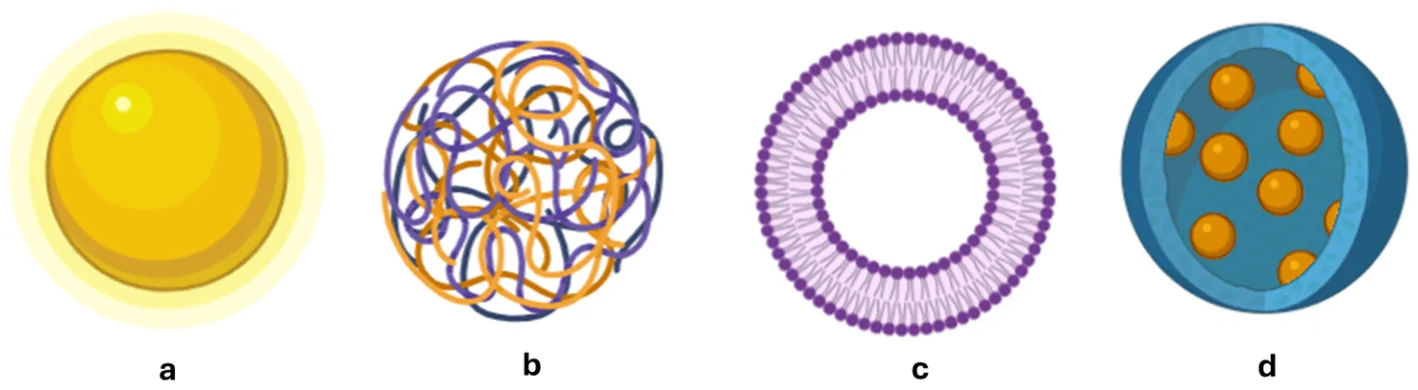
Representative nanoscale configurations used in antimicrobial and drug delivery applications: (**a**) metallic nanoparticle, (**b**) polymeric nanoparticle, (**c**) liposome and (**d**) drug-loaded nanoparticle.

Nanomaterials offer several advantages over their bulk counterparts, including high surface-to-volume ratios, enhanced interaction with biological targets, and increased drug-loading capacity. At the nanoscale, materials present tunable surface chemistry, improved binding control, and size-dependent physiochemical effects (such as plasmonic behaviour), which can be leveraged for imaging, diagnostics and therapeutics [65,66,67]. Nanoparticles (NPs) can be precisely engineered in size and composition to enhance cellular uptake and enable use-inspired drug release kinetics, making them particularly attractive for antimicrobial applications [22,68]. A broad range of nanomaterial architectures has been developed from organic compounds, metals, and natural and synthetic polymers to meet the requirements of the specific biomedical applications. Relevant configurations of antibacterial nanomaterials are shown in Figure 1.

A variety of naturally occurring substances possess antimicrobial properties that can be enhanced through nanoscale formulation. For example, curcumin nanoformulations have been reported to be more effective at treating infections than their macroscale variants, especially when tested in conjunction with a metal phase. Curcumin-loaded Zn, Ag, Fe and Cu NPs have been described as effective antimicrobial agents, including in hybrid systems that combine organic bioactives with metal-based scaffolds [69,70,71,72]. At the same time, emerging evidence suggests that prolonged or sub-lethal exposure to metal-based nanoparticles may induce adaptive stress responses or tolerance in bacteria, highlighting the need for careful consideration of resistance risks, which are discussed further in Section 4.

AgNPs are among the most extensively investigated nanomaterials against MDR pathogens. They have shown efficacy against both Gram-positive and Gram-negative bacteria, even though Gram-positive strains can be less susceptible due to the thicker peptidoglycan layer (ca. 20–80 nm) and associated surface charge characteristics [73]. The antimicrobial activity of AgNPs is commonly attributed to Ag ion release, membrane disruption, increased permeability, intracellular penetration, and subsequent damage to essential cellular components. AgNP-associated ROS generation has also been reported [74], although the relative contribution of individual mechanisms remains under debate [75]. Ntolia and colleagues investigated the antibacterial activity of negatively charged AgNPs encapsulated in collagen-based gels and found that 6–10 nm particles exhibited broad-spectrum antimicrobial activity against both Gram-positive and Gram-negative bacteria (MIC 10–20 ppm) [76]. Likewise, Shaaban et al. reported strong antibacterial activity of AgNPs against a range of pathogens (*S. aureus*, *P. aeruginosa*, *S. typhi*, and *E. coli*) [77].

Other than Ag, AuNPs offer distinct advantages linked to the physiochemical stability and surface chemistry of gold, which enable functionalisation with diverse ligands via multivalent surface interactions [78]. Since antimicrobial activity is influenced by particle size, morphology, and surface chemistry [79], AuNPs have been produced in different geometries (e.g., nanorods, nanoshell, nanocluster and nanostars) [80,81], with several studies reporting improved antimicrobial efficiencies on shaped constructs [82]. AuNPs have therefore been proposed as promising candidates for addressing AMR-associated infections [83], whereby combination strategies have been reported to further enhance performance. For example, AuNPs in combination with chitosan have been reported to exert synergistic effects against *P. aeruginosa* and *S. aureus* [84]. Another study compared AuNPs fabricated via biological (using ginger and curcumin) and chemical methods and found that biologically formulated AuNPs outperformed the chemically produced variants against *P. aeruginosa*, *E. coli*, and *S. aureus* [85].

CuNPs, including nanosized particles (CuONPs), are also widely explored as candidates to control and reverse the spread of AMR. Their antimicrobial action is understood to follow mechanisms comparable to those of other metal NPs, i.e., release of ions and respective interaction with bacteria [86,87]. Lai and colleagues evaluated the antimicrobial activity of CuNPs of two different sizes (20 and 60 nm) across a concentration range of 1–100 μg/mL, reporting higher efficacy for the 20 nm particles at lower concentrations and multiple antimicrobial mechanisms, including membrane disruption and DNA damage [88]. Another study investigated lipopeptide-stabilised CuONPs (⌀: 30  ±  2 nm) against Gram-positive *B. subtilis* CN2 strains and Gram-negative *P. aeruginosa* CB1 strains. Antibacterial activity and significant ROS generation was observed against both pathogens, supporting the potential of these systems in biocompatible bactericidal applications [89].

Synergistic combinations of metal-based NPs (for example, Cu-Ag, and Ag-Au) have also been investigated and reported to enhance antimicrobial efficacy compared to single-metal systems [90,91]. Beyond Ag, Au and Cu, nanoparticles based on Zn, cobalt, titanium and silicon have also shown antimicrobial potential and are discussed in detail in other reviews [92,93,94,95].

### 2.3. Polymeric Antimicrobial Nanomaterials

Following administration, conventional drugs undergo absorption, distribution, metabolism and excretion (ADME) through the bloodstream. In this process, a considerable amount of the active pharmaceutical ingredient (API) undergoes rapid clearance and inactivation before reaching the target site. Polymeric nanosystems aim to address these challenges by improving drug solubility, stability, and bioavailability, while enabling controlled and site-directed release [96,97]. Polymeric nanosystems encompass a broad class of polymer-based nanocarriers with diverse internal architectures (Figure 1); among these, matrix-type nanospheres are solid polymeric particles with uniformly dispersed antimicrobial agents; polymer–metal hybrid nanoparticles integrate metallic antimicrobial components within polymer matrices or shells to enable multi-modal activity. For antimicrobial applications, polymers of either natural or synthetic origin can also be formulated into core–shell nanocapsules, liposomes, dendrimers, and nanocomposites [98].

**Natural polymers.** Among natural polymers, chitosan is one of the most widely studied, due to its intrinsic antimicrobial properties, either alone or in combination with other agents. While chitosan is traditionally produced from crustacean-derived chitin isolated from the shells of shrimps and other molluscs, recent studies have indicated that fungal sources (mushrooms) may provide comparable utility across applications [99]. Several factors influence chitosan’s antimicrobial activity; including pH, molecular weight, degree of acetylation, chemical modification, concentration, metal complexation, and the bacterial strain [100,101,102]. While many studies indicate greater susceptibility of Gram-negative bacteria compared to Gram-positive bacteria, conflicting findings have also been reported [103]. Proposed mechanisms of chitosan antimicrobial activity include disruption of the cell wall/membrane, formation of a dense polymer layer on the bacterial surface, electrostatic interactions with microbial DNA, and chelating effects [104]. Beyond chitosan, ε-polylysine is another natural polymer that has been explored in antimicrobial nanoparticle-based systems and antimicrobial peptide-inspired surface coatings. Its intrinsic antimicrobial activity is primarily attributed to its cationic, membrane-active behaviour, and this favourable safety profile has underpinned the approval of ε-polylysine as a food preservative since the early 2000s [105,106]. Natural polymers, such as chitosan, are often combined with metal or other materials to achieve a synergistic antimicrobial performance. For example, chitosan NPs loaded with cerium oxide at concentrations of 10, 20 and 30 wt.% inhibited the growth of *S. aureus* and *E. coli* strains [107]. Negligible cytotoxic effects were observed when the same nanohybrids were evaluated using bone marrow mesenchymal stromal/stem cells (BM-MSCs), supporting their potential for treating bone-related infections. In another study in vitro, chitosan NPs loaded with Fe^2+^ or Fe^3+^ ions exhibited higher antimicrobial activity against *E. coli*, *S. aureus* and *C. albicans*, compared to their iron-free counterparts, with Fe^2+^-loaded systems showing superior performance [108]. Finally, Ahmad et al. designed chitosan derivatives to mimic the amphiphilic topology of AMPs. Chitosan grafted with both arginine and oleic acid (CH-Arg-OA) demonstrated strong antimicrobial activity against Gram-negative bacteria and was well tolerated by both HEK293 and HepG2 cell lines, highlighting its potential for antimicrobial applications [109].

**Figure 2:**
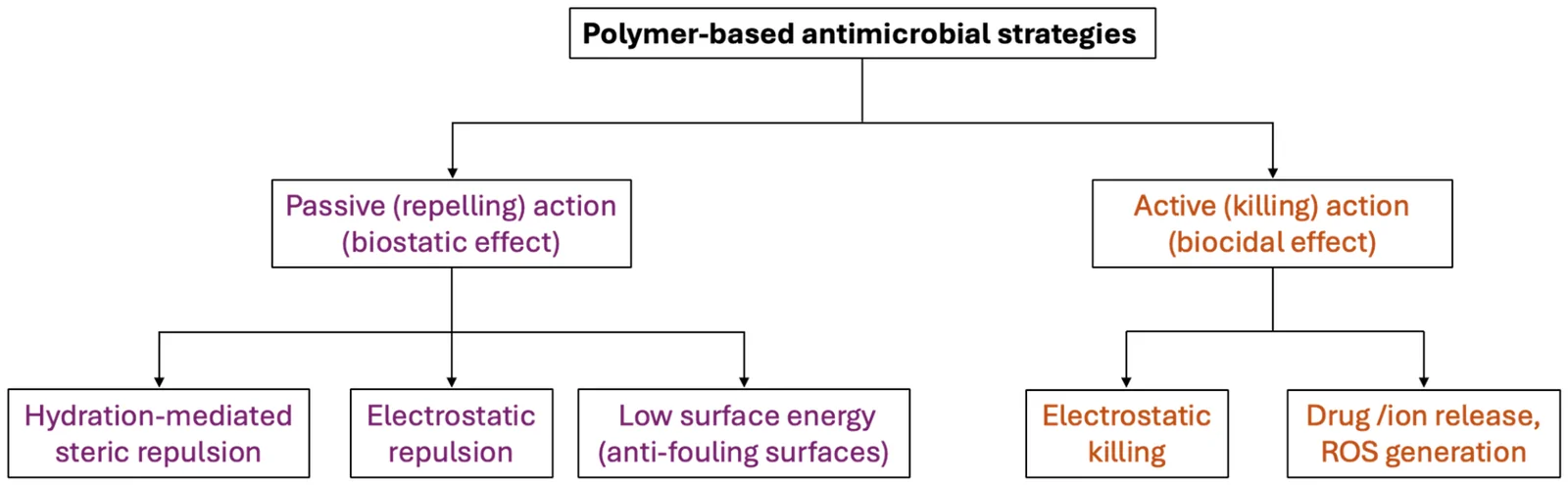
Classification of polymer-based antimicrobial surface strategies according to biostatic and biocidal effects.

**Synthetic polymers.** Synthetic polymers are another important class of materials that can be engineered to exhibit antimicrobial activity through either biostatic (passive) or biocidal (active) effects, as presented in Figure 2. Biostatic effects originate from polymer surface chemistry and act to reduce bacterial adhesion and minimise biofilm formation. Biocidal effects lead to microbial disruption through direct interaction with the polymer surface or via surface-mediated release of antimicrobial agents [110]. Polymer surfaces can be functionalised to equip medical devices (e.g., hygiene products, implants, and regenerative scaffolds) with tailored antimicrobial activity based on the intended application. Common strategies include chemical grafting of positively charged functional groups (e.g., quaternary ammonium cations), surface coating (layer-by-layer), plasma treatment, nanoparticle encapsulation, micro/nano patterning and drug loading (Table 1). These strategies are reviewed in detail elsewhere [111,112]. Among cationic polymers, polylysines have attracted significant interest because their antimicrobial activity is primarily associated with membrane-targeting mechanisms that are considered less prone to inducing classical antibiotic resistance [113,114]. These polymers typically present cationic moieties that promote adsorption to negatively charged microbial membranes, and hydrophobic segments that insert into the lipid bilayer of the cell membrane, leading to structural disruption and cell death [115].

Liu et al. compared six cationic polymers, including poly(hexamethylene guanidine) (PHMG), polyethyleneimines of different molecular weights, α-polylysine, ε-polylysine, and polyacrylamide, against persistent subpopulations of *MRSA*, *MSSA* and *E. coli*, reporting PHMG as the most effective candidate (Minimum Bactericidal Concentration or MBC 2 μg/mL) [116]. Polyhexamethylene biguanide (PHMB) is another cationic polymer that is known to disrupt bacterial membranes and induce cell death [117]. In comparative testing against mastitis-associated strains, PHMB and PHMG reduced microbial load by more than 90% relative to benzalkonium chloride, supporting their potential in veterinary infection control applications [118].

### 2.4. Metal–Organic Frameworks (MOFs)

MOFs are a class of nanoporous materials composed of metal ions (or clusters) coordinated to organic ligands. They have attracted significant attention for biomedical applications due to their tunable porosity, high drug-loading capacity, and reported antibacterial potential [119]. MOFs can be tailored through control of metal centres, linker chemistry, and framework topology, enabling use-inspired functionalities. Based on composition, respective systems may incorporate one (single-metal), two (bimetallic), or multiple metals, with opportunities for synergistic activity arising from combined mechanisms and multifunctionality [120,121].

Other than molecular and microscale factors, MOF performance can also be tuned by variations in particle size, surface-to-volume ratio and drug release capability relevant to antimicrobial interactions [122]. Illustrative examples include a porphyrinic bimetallic MOF, i.e., PCN-224(Zr/Ti), incorporated into poly(lactic-*co*-glycolic acid) nanofibres for wound-dressing applications, where the presence of Ti enhanced ROS generation and photodynamic antimicrobial capability, enabling antibiotic-free activity against MDR bacteria [123]. Other MOFs relevant for antibacterial strategies include Zeolitic imidazolate framework-8 (ZIF-8); for instance, Ag-doped γ-Fe_2_O_3_@SiO_2_@ZIF-8-Ag (FSZ-Ag) was reported to exhibit strong bactericidal activity against both Gram-negative *E. coli* and Gram-positive *S. aureus* [124]. In addition, Zn- and Co-based MOFs have been reported to display antibacterial, antioxidant and antibiofilm activity, with differing profiles attributed to variations in porosity/surface area and redox behaviour [125].

**Figure 3:**
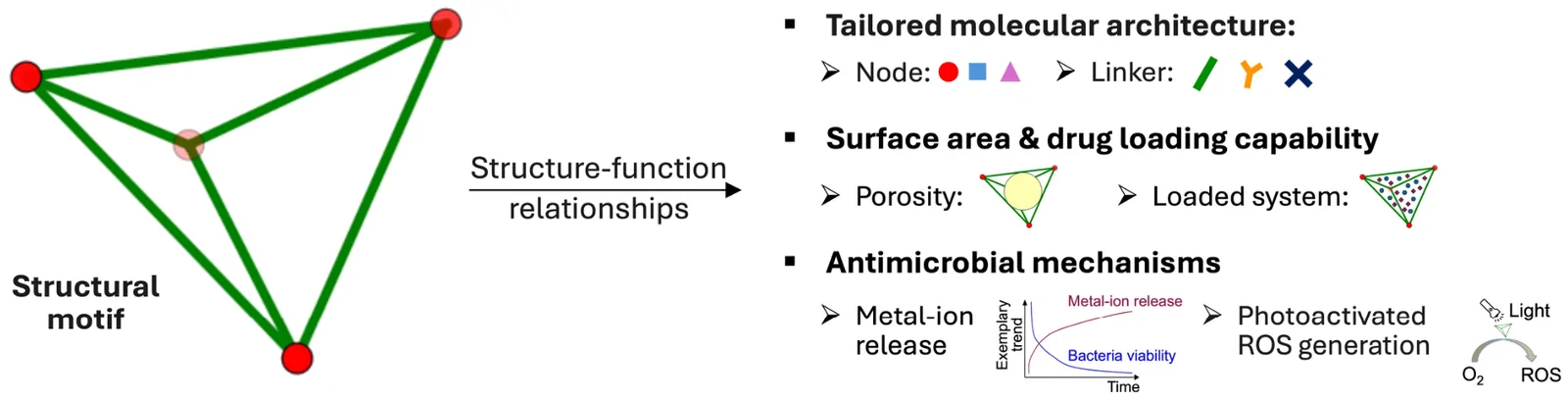
Schematic illustration of a ZIF-8-type metal–organic framework, highlighting tetrahedral metal–ligand coordination and the resulting porous cage architecture. Variation in node and linker valency generates tailored molecular architectures, while the high internal surface area enables efficient porosity-driven drug loading. Antimicrobial activity can arise from metal-ion release or external stimuli (e.g., photoactivated reactive oxygen species generation). Metal-ion release and bacterial viability profiles are illustrated as an exemplary qualitative relationship. This plot represents a conceptual schematic of the mechanism.

Mechanistically, MOF-associated antibacterial effects have been attributed to metal-ion release, ROS generation mediated by metal nodes, organic ligands, or both, and, in some systems, contact-mediated membrane damage. Selected mechanisms are conceptually represented in Figure 3. The presence of multiple antimicrobial pathways is relevant as it may reduce the likelihood of resistance development comparable to single-mechanism agents. However, susceptibility can vary by organism, with several studies suggesting that Gram-negative bacteria may be more susceptible to MOFs than Gram-positive bacteria [126].

The high porosity of MOFs can be leveraged to achieve significant drug loading , while the incorporation of multiple metal ions may further enhance bactericidal activity through cooperative antimicrobial mechanisms (Table S1, Supplementary Information). Nevertheless, systematic cytotoxicity evaluation remains essential to enable biomedical applicability. Table 2 provides a brief comparison of the structural and antimicrobial characteristics of MOFs, AMPs and antimicrobial proteins. Representative examples are provided in Tables S1 and S2 (Supplementary Information), respectively. Overall, MOFs typically offer greater structural tunability and drug-delivery capability, whereas biological antimicrobials often provide greater biological specificity and intrinsic antimicrobial activity.

### 2.5. Stimulus-Responsive Antimicrobial Strategies

**Figure 4:**
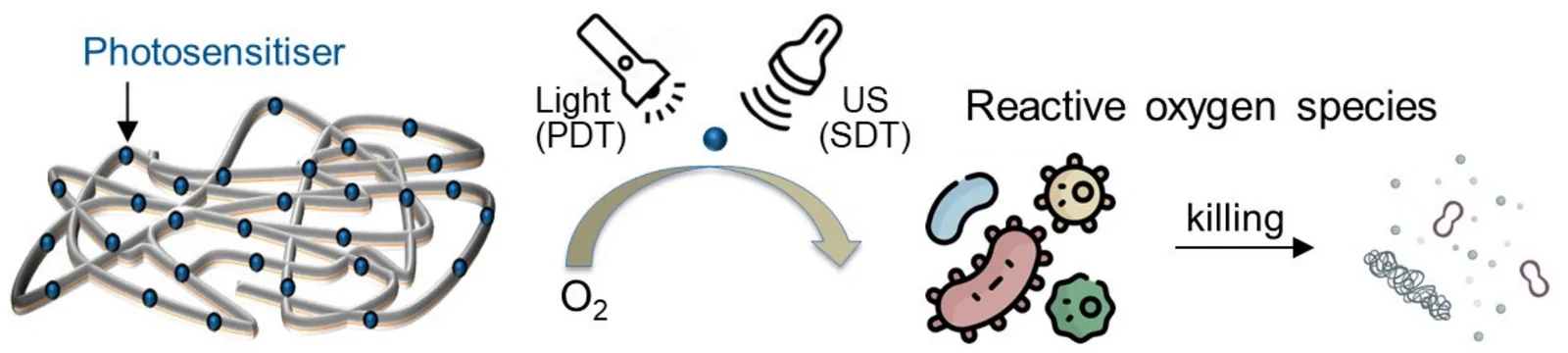
Schematic illustration of stimulus-responsive antimicrobial strategies based on photodynamic therapy (PDT) and sonodynamic therapy (SDT). Upon light or ultrasound stimulation of a sensitiser-loaded polymer system in the presence of molecular oxygen, reactive oxygen species (ROS) are generated, leading to multi-target oxidative damage of bacterial membranes and intracellular components, and, ultimately, to bacterial killing.

Stimulus-responsive antimicrobial strategies have emerged as a promising approach for precise and localised infection control, offering potential advantages over conventional antimicrobial therapies , including reduced systemic drug exposure, improved therapeutic efficacy and minimisation of off-target effects. Integration of antimicrobial agents with stimulus-responsive polymers enables localised, on-demand antimicrobial activity in response to specific environmental triggers, such as light, ultrasound (US), pH, bacterial enzymes or oxidative stress. Photodynamic therapy (PDT), photodynamic materials [127,128,129,130,131], as well as sonodynamic therapy (SDT) [132,133,134] are particularly promising because they leverage molecular oxygen and exposure to light and ultrasound, respectively, to achieve ROS generation and subsequent irreversible damage of the internal components of bacteria, e.g., lipids, proteins, and nucleic acids. Unlike traditional antibiotic approaches that rely on specific molecular targets, these multi-target ROS-mediated antimicrobial pathways are broad, rapid, and non-specific, making them less susceptible to the rapid emergence of resistance observed with many target-specific antibiotics. Control of photosensitiser dosage and stimulus exposure parameters can also be leveraged to minimise off-target cytotoxicity (Figure 4).

The potential of stimulus-responsive antimicrobial technologies lies in their ability to circumvent the evolutionary pathways that drive antimicrobial resistance. PDT and SDT are especially valuable because their ROS-mediated killing mechanisms do not depend on antibiotic binding sites, efflux susceptibility, or enzymatic degradation, making them effective even against multidrug-resistant pathogens.

The spatially controlled activation of stimulus-responsive antimicrobial technologies also reduces collateral damage to host tissues and preserves the commensal microbiome, addressing key limitations of systemic antibiotics [135,136]. Emerging work combining PDT or SDT with nanocarriers, oxygen-generating materials, or biofilm-penetrating agents further enhances their efficacy in hypoxic or biofilm-rich environments where antibiotics typically fail. As these technologies become more tunable, biocompatible, and clinically validated, they offer a promising complementary route to reduce antibiotic reliance and mitigate the burden associated with AMR [137,138].

Cui and colleagues developed a composite antibacterial hydrogel doped with polymer NP to achieve combined PDT and photothermal therapy (PTT) antibacterial capabilities. Their data indicated that when the composite was sequentially irradiated with white and NIR light, synergistic PDT and PTT mechanisms yielded stronger antibacterial effects than either alone [139]. Building on this research, Ag-based photoactivated antibacterial systems have also been developed, aiming to fight drug-resistant bacterial infections. Polymeric micelles based on amphiphilic poly(aspartic acid)-block-poly(ε-caprolactone) (PAspb-PCL) were used as nanocarriers to encapsulate protoporphyrin IX (PpIX), a well-known photosensitiser, within the micellar core. This complex was then decorated with AgNPs on the micellar shell through a reduction method in situ. Their data in vitro suggested enhanced antimicrobial activity of the complex compared to monotherapy, while in vivo studies demonstrated effective eradication of drug-resistant *S. aureus* in a murine model [140]. Liu et al. fabricated lecithin bilayer-coated poly(lactic-co-glycolic) NP preloaded with verteporfin (Ver-PLGA@Lecithin) to mediate SDT against *H. pylori* infection. The team found that when combined with ultrasound, Ver-PLGA@Lecithin inactivated *H. pylori* via ROS production. The approach also demonstrated therapeutic efficacy comparable to standard triple therapy in a mouse model [141].

### 2.6. Biological and Bioinspired Antimicrobial Strategies

Macrocycles such as cyclic peptides, cyclodextrin derivatives, and synthetic macrocyclic scaffolds have gained attention for their ability to disrupt bacterial membranes, chelate essential ions, or inhibit protein–protein interactions [142,143]. Their pre-organised and cyclic structures enhance binding affinity and stability, as well as their specificity, making them effective against multidrug-resistant (MDR) pathogens. Calixarenes and related macrocycles have been investigated as drug delivery vehicles, host-guest antimicrobial systems and as molecular prodrugs. These have also shown antimicrobial activity since the 1950s, with recent work highlighting macrocyclic antibiotics and synthetic macrocycles possessing potent activity against MRSA, *Pseudomonas*, and biofilms [144,145].

AMPs and their synthetic mimics remain one of the most versatile antibiotic-free platforms owing to their borad mechanisms of action [140,146]. Many naturally occurring AMPs also possess cyclic or constrained architectures, illustrating the conceptual overlap between macrocyclic and peptide-based antimicrobial strategies. AMPs are generally short, cationic, amphipathic molecules that exert antimicrobial activity through multiple mechanisms. Their primary mode of action involves electrostatic interactions with negatively charged microbial membranes, resulting in membrane permeabilisation, pore formation, and subsequent cell death. Their amphipathic structures enable rapid membrane disruption, while some also modulate host immunity or inhibit biofilms. Synthetic and engineered AMPs have improved stability, reduced cytotoxicity, and demonstrated broad-spectrum activity against MDR Gram-positive and Gram-negative bacteria [147]. Recent studies highlight peptide mimetics, D-amino-acid analogues, and polymer–peptide hybrids with enhanced resistance to proteolysis [148,149]. Despite these advantages, several challenges continue to limit clinical translation, including proteolytic degradability, short half-life and reduced activity under physiological conditions. Furthermore, potential cytotoxicity, haemolytic activity, manufacturing costs, and delivery challenges are still barriers to their widespread therapeutic use.

Antimicrobial antibodies are another approach that is receiving growing attention in the battle against AMR. These antibodies offer highly specific antimicrobial activity by a myriad range of mechanisms that include neutralisation of toxins, inhibition of toxin secretion, blocking adhesion, or promoting opsonophagocytic killing [150,151]. Growing evidence supports the use of antibodies as adjunctive or preventive therapies for bacterial infections. One of the most successful examples is bezlotoxumab, which neutralises *Clostridioides difficile* toxin B and reduces recurrence of infection [152]. Monoclonal antibodies targeting *S. aureus*, *P. aeruginosa*, and *C. difficile* have shown promise in reducing bacterial burden and preventing recurrence, particularly when combined with standard therapies [153,154]. Advances in Fc-engineering and bispecific formats further enhance their potency and tissue penetration [155]. This high specificity of antibody therapeutics minimises off-target effects and microbiome disruption, although it necessitates accurate pathogen identification prior to administration. Antigenic variability, immune evasion mechanisms, restricted tissue penetration, and reduced effectiveness in immunocompromised individuals may also limit therapeutic success. Furthermore, production costs and the requirement for parenteral administration remain challenging for widespread implementation.

Bacteriophages are viruses that specifically infect bacteria and represent one of the most extensively investigated alternatives to antibiotics. Therapeutic applications primarily utilise lytic phages, which bind specific receptors on the bacterial surface, inject their genetic material and replicate within the host bacterium, ultimately inducing bacterial lysis. This self-replicating property distinguishes phages from conventional antimicrobials because phage numbers can increase at the site of infection as long as susceptible bacterial hosts remain available. Bacteriophages and phage-derived enzymes provide highly targeted antimicrobial activity with minimal impact on the microbiome [156,157]. These can self-amplify at infection sites, penetrate biofilms, and evolve alongside bacterial hosts, offering a potential, dynamic countermeasure to AMR [158,159,160]. Phage lysins are enzymes that degrade bacterial cell walls, showing rapid bactericidal activity and efficacy against MDR pathogens, including MRSA and VRE [161]. Several limitations continue to constrain broader implementation. Phages often possess narrow host ranges, requiring careful matching to the infecting bacterial strain. Bacterial resistance to phages can emerge, while host immune responses may reduce phage persistence following repeated administration. Additional concerns include variability in pharmacokinetics, difficulties in standardising personalised phage preparations, and regulatory challenges associated with biological entities capable of evolution and adaptation.

EVs are lipid bilayer-enclosed nanoparticles naturally released by both eukaryotic and prokaryotic cells. EVs, including exosomes and microvesicles, have emerged as promising antibiotic-free antimicrobial agents due to their ability to modulate host immunity, disrupt bacterial viability, and inhibit biofilm formation [162,163]. The antimicrobial effects of EVs arise through several mechanisms, including delivery of antimicrobial peptides and enzymes, modulation of innate and adaptive immune responses, neutralisation of microbial toxins, enhancement of bacterial clearance, and promotion of tissue repair. MSC-derived EVs carry a rich cargo of antimicrobial peptides (e.g., LL-37), microRNAs, cytokines, and metabolic enzymes that enhance phagocytic activity, promote neutrophil recruitment, and suppress bacterial growth [164]. Several studies demonstrate that MSC-derived EVs can directly impair bacterial membrane integrity, reduce virulence factor expression, and attenuate quorum-sensing pathways, offering a multi-target mechanism that is difficult for pathogens to evade [165,166]. Their nanoscale size also enables penetration into biofilms, where they can weaken extracellular polymeric substances and enhance susceptibility to host defences. Importantly, MSC-EVs exhibit low cytotoxicity, strong biocompatibility, and immunomodulatory benefits, making them attractive candidates for treating chronic wounds, sepsis, and device-associated infections [167]. However, the translation of EV therapeutics is challenged by significant biological and technical considerations. EV preparations are frequently heterogeneous, and differences in isolation methods, source materials, culture conditions, and purification protocols can profoundly influence composition and function. Additional concerns include scalability, batch-to-batch reproducibility, storage stability, biodistribution, immunogenicity, and a rapidly evolving regulatory landscape.

### 2.7. AI-Assisted Antimicrobial Discovery and Design

AI is increasingly transforming the discovery and optimisation of antimicrobial materials and biomolecules, particularly AMPs, by reducing the time and cost associated with conventional screening. Deep learning, generative models, and protein-language models can now identify sequence patterns associated with antimicrobial activity, predict toxicity and hemolysis, and generate entirely new peptide candidates for experimental validation [168]. For example, Luo and colleagues developed machine learning (ML) workflows that outperform traditional trial-and-error approaches in biomaterial design [169]. Recent generative AI platforms have successfully produced novel AMPs with potent activity against multidrug-resistant pathogens including MRSA and carbapenem-resistant *Acinetobacter baumannii*. Experimental validation of AI-designed peptides has demonstrated that computationally generated candidates can retain strong antimicrobial efficacy while minimising host toxicity, highlighting the growing maturity of AI-assisted biomolecular design [170]. In parallel, deep learning-guided peptide generation combined with high-throughput synthesis and screening has enabled the rapid identification of previously unexplored antimicrobial sequence space, accelerating translation from in silico prediction to in vitro and in vivo testing.

For antimicrobial MOFs, AI adoption is at an earlier but rapidly advancing stage. Recent machine learning models can predict MOF crystal structures, synthesis conditions, and physicochemical properties from large databases, thereby accelerating the identification of candidates with desirable antimicrobial functions such as controlled metal-ion release, reactive oxygen species generation, and drug-loading capacity. Data-driven approaches have been used to automatically mine synthesis information from the literature and predict experimental conditions for new MOFs, outperforming expert intuition in some cases [171]. More recently, large language model-enabled platforms such as ChatMOF have demonstrated the capability to predict and generate MOF structures through natural-language interaction, opening opportunities for inverse design of antimicrobial MOFs tailored to specific pathogens or biomedical applications [172]. Nevertheless, significant challenges remain, including limited high-quality antimicrobial datasets, lack of standardised biological testing protocols, difficulties in linking MOF structure directly to antimicrobial performance, and the need for experimentally validated AI predictions. Future progress will depend on integrating generative AI, automated synthesis, robotics, and biological screening to create closed-loop discovery systems capable of rapidly developing safe and effective antimicrobial MOFs and biomolecular therapeutics [173].

### 2.8. Design Principles for Antibiotic-Free Antimicrobial Materials

Despite the diversity of antibiotic-free antimicrobial strategies described in the previous sections, a set of shared design principles can be identified across material classes and therapeutic approaches. These principles provide a conceptual framework to guide the rational development of next-generation antimicrobial systems that are effective, biocompatible, and resilient to AMR. Unlike conventional antibiotics, which typically rely on highly specific molecular targets, antibiotic-free strategies are predominantly governed by physicochemical interactions, multi-modal mechanisms, and adaptive material responses. The present section focuses on overarching design considerations that are broadly applicable across antimicrobial platforms, rather than on quantitative structure–property–performance relationships, which are often highly material- and application-specific. The key design principles are outlined below.

**Multi-target antimicrobial mechanisms.** A central principle of antibiotic-free strategies is the use of multi-target antimicrobial mechanisms that reduce the likelihood of resistance development [21,22,135]. Unlike conventional antibiotics, many antibiotic-free systems, including metal nanoparticles, antimicrobial peptides, and photodynamic/sonodynamic platforms, operate through simultaneous disruption of multiple cellular components, such as membranes, proteins, nucleic acids, and metabolic pathways. The combination of these effects produces a broad-spectrum mode of action that is significantly more difficult for microorganisms to evade. Such multi-target activity can be achieved through synergistic material design, whereby complementary antimicrobial mechanisms are integrated within a single system. Examples include nanoparticles embedded within polymer matrices, MOFs loaded with antimicrobial agents [119,120,121,122], or combined photodynamic and photothermal platforms, which can achieve greater performance than individual components alone.

**Stimulus-responsiveness.** Another key principle is the incorporation of stimulus-responsive functionality, enabling precise control over the timing and location of antimicrobial activity. Environmental stimuli may originate from externally applied triggers, such as light, ultrasound or temperature changes, or from infection-associated biochemical cues, including pH shifts, hypoxia and enzymatic activity. Stimulus-responsive systems can therefore activate antimicrobial effects in a spatiotemporally controlled manner, minimising off-target toxicity and improving therapeutic efficiency [26,27,28,127]. For instance, photodynamic and sonodynamic therapies rely on external activation to generate ROS, while thermosensitive polymers can release antimicrobial agents selectively in inflamed microenvironments. This approach enables on-demand antimicrobial action, reducing unnecessary exposure and potentially limiting the emergence of resistance. Microenvironment-responsive materials are engineered to exploit local infection-associated biochemical changes to enhance penetration, retention, and therapeutic efficacy [137,138,141]. Examples include (i) oxygen-generating systems that overcome hypoxia in PDT; (ii) enzyme-responsive carriers that degrade in the presence of bacterial enzymes; (iii) nanomaterials capable of penetrating biofilm structures. By tailoring material properties according to specific biochemical signals, it is possible to achieve improved targeting and enhanced antimicrobial performance in complex infection settings.

**Host tolerability.** A critical design consideration in antibiotic-free materials is maintaining host tolerability while preserving antimicrobial potency. Effective antimicrobial mechanisms, such as ROS generation [174], metal-ion release [175], and membrane disruption [176], can also induce cytotoxicity, inflammation, or tissue damage if not carefully controlled. Other than raw material selection, controlled therapeutic dosage, targeted activation and surface functionalisation can be leveraged to minimise systemic exposure and enhance local efficacy.

**Translational feasibility.** The impact of antibiotic-free antimicrobial strategies relies on their translational feasibility, including manufacturability, regulatory compliance, and cost-effectiveness. Many advanced materials demonstrate promising antimicrobial activity at the laboratory scale but face challenges in scaling up or meeting regulatory requirements. Key considerations in the material design stage include industry-compliant manufacturability, durability, and compatibility with existing medical devices and clinical workflows [66,67,94]. Incorporating these factors at the design stage is essential to bridge the gap between proof-of-concept and real-world applications, ensuring effective technology translation into clinical and industrial use.

## 3. Application of Antibiotic-Free Antimicrobial Approaches

### 3.1. Bone Regeneration and Repair

**Figure 5:**
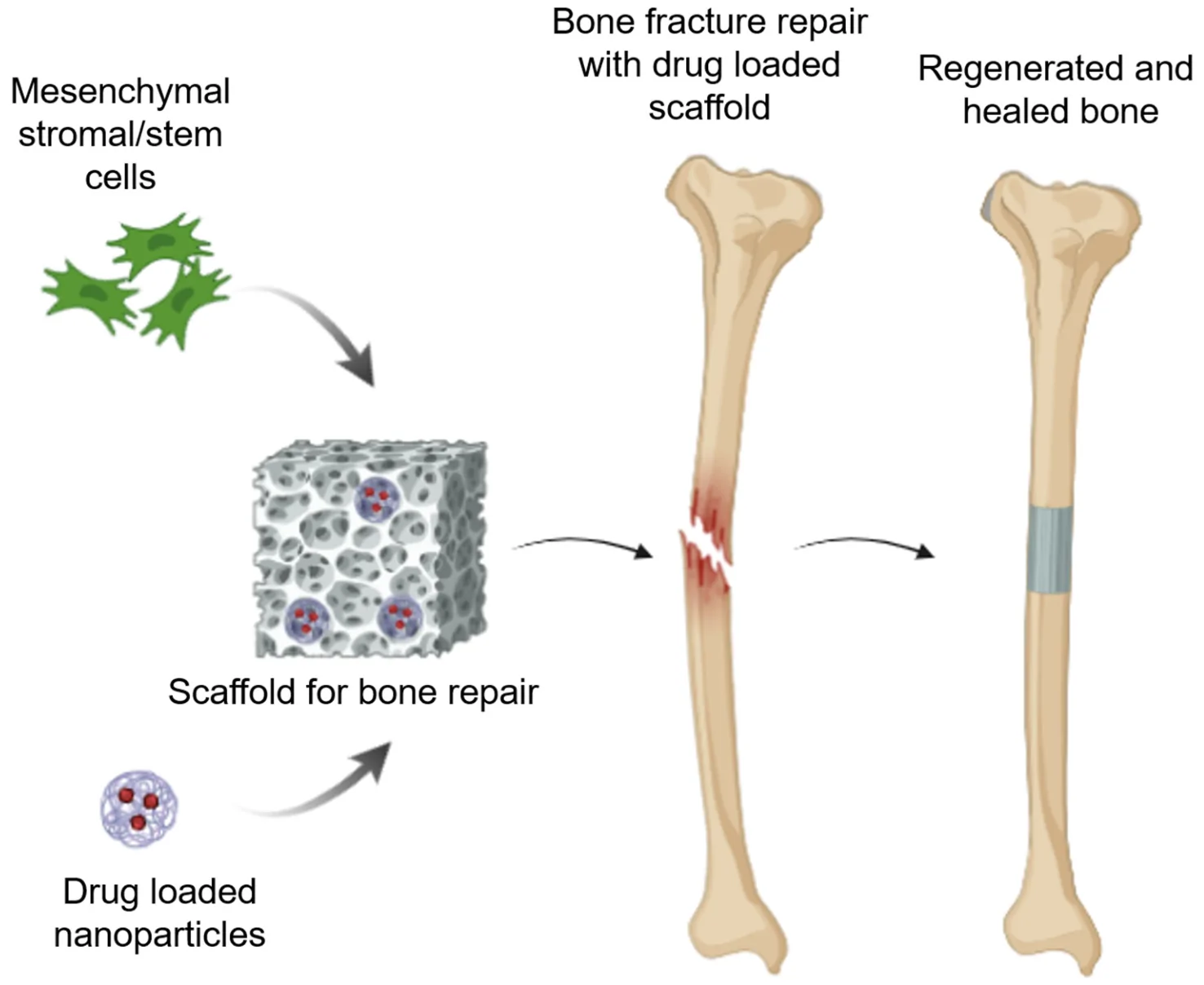
Representative nanoparticle-based scaffold configurations combining antimicrobial and osteogenic functionalities for bone repair and regeneration.

Recent advances in antimicrobial chemistries and orthopaedic surgery have accelerated the development of antibiotic-free, material-driven approaches to prevent and treat bone infections, particularly those associated with implants and chronic osteomyelitis [177]. Ion-releasing coatings incorporating zinc, copper, gallium, or strontium on titanium and bioactive glass surfaces provide simultaneous antibacterial and osteogenic effects without relying on silver or antibiotics [178,179]. Polymer-based approaches, including peptide-loaded hydrogel composites and photodynamic nanomaterials, can trigger antibacterial activity as well as anti-inflammatory function to support a pro-healing environment [180,181]. Stimulus-responsive systems enabling photodynamic, photothermal, and sonodynamic therapies enable on-demand bacterial eradication within bone defects or around implants (Figure 5), showing strong efficacy against MRSA biofilms while supporting bone regeneration [182]. Several antibiotic-free biomaterial platforms have been investigated, including cerium oxide NP-loaded chitosan scaffolds [107], chlorhexidine-containing bone cements [183], and metal–hydroxyapatite NPs [184]. Encapsulation of antimicrobial peptides in polymer scaffolds and using nanoscale approaches have also been proposed to combat bone infection and promote bone regeneration [185].

Biological strategies have also gained traction, with bacteriophages and MSC-derived EVs demonstrating potent activity against *S. aureus* and other bone-associated pathogens [186,187]. Phage-loaded hydrogels and lysin-functionalised implants show strong biofilm disruption and improved healing in osteomyelitis models [188,189]. Likewise, antimicrobial peptides grafted onto titanium or incorporated into scaffolds provide contact-killing and immunomodulatory functions while maintaining osteogenic compatibility [190,191]. Together with nanoscale implants and 3D-printed scaffolds loaded with ion-releasing and photothermal agents, these systems offer multifunctional platforms capable of simultaneously addressing infection control and skeletal repair.

The use of coated nails for fracture-related infections (FRIs), such as commercial products like the ETN PROtect and the ZNN Bactiguard, have been recorded to provide higher protection from infections compared to standard noncoated nails in clinical scenarios with high FRI risk [192]. Bacteriophage therapy, as discussed in Section 2.6 above, is being investigated for personalised approaches in combination with antibiotics in musculoskeletal infections [193].

### 3.2. Chronic Wounds and Skin Infections

Infection is one of the leading causes of morbidity, delayed healing, disability, and mortality in chronic wounds. Non-self-healing skin injuries, such as diabetic and pressure ulcers, generate large, open areas of devitalised tissue that are particularly susceptible to bacterial colonisation and biofilm formation. In the case of diabetic foot ulcers alone, estimates indicate that approximately 60% are infected on presentation and that around 20% of moderate-to-severe infections progress to lower-limb amputation [194,195]. This incidence is expected to increase further due to the rising prevalence of multidrug-resistant pathogens, the slow pace of regulatory approval for new systemic antibiotics, and growing environmental and safety concerns associated with metal-based, specifically silver [196], antimicrobial chemistries. Antibiotic-free infection-control strategies for chronic wound care are therefore urgently needed in community and homecare settings to minimise reliance on skilled clinical personnel and reduce the burden on healthcare systems.

Manuka honey-containing textile materials [197] and methylglyoxal-containing fibres [198] have been investigated as antibiotic-free antimicrobial systems for wound management. In parallel, stimulus-responsive materials offer an attractive strategy for the controlled activation of antimicrobial function. In particular, photodynamic polymer-based systems, in which antimicrobial activity is activated on demand via controlled light exposure, are well suited for decentralised care models due to the direct accessibility of cutaneous wounds. Such materials may enable prompt, localised antimicrobial action and tissue repair, while minimising unnecessary or prolonged exposure to antibiotics or metal-based antimicrobials, thereby reducing selective pressures associated with antimicrobal resistance [199]. Notably, the use of low-cost and widely available light sources, including ambient light, supports these approaches and aligns with current trends toward home-based wound care. For instance, two-hour sunlight exposure of L-arginine-modified zinc oxide (ZnO) nanoparticles was found to trigger more than 99% killing of *S. aureus*, *E. coli* and *MRSA*, while maintaining minimal cytotoxicity [200]. In vivo testing of the corresponding particle-encapsulated polyvinyl alcohol-based hydrogel in a rat model of full-thickness *S. aureus*-infected skin defects demonstrated reduced bacterial colonisation and promotion of angiogenesis and collagen deposition towards effective repair of infected wounds. Leveraging this work, a sunlight-activated nanospray formulation consisting of a new photosensitiser encapsulated in chitosan oligosaccharide-coated nanoparticles achieved rapid and effective management of diabetic wounds in outdoor and resource-limited settings [201].

**Figure 6:**
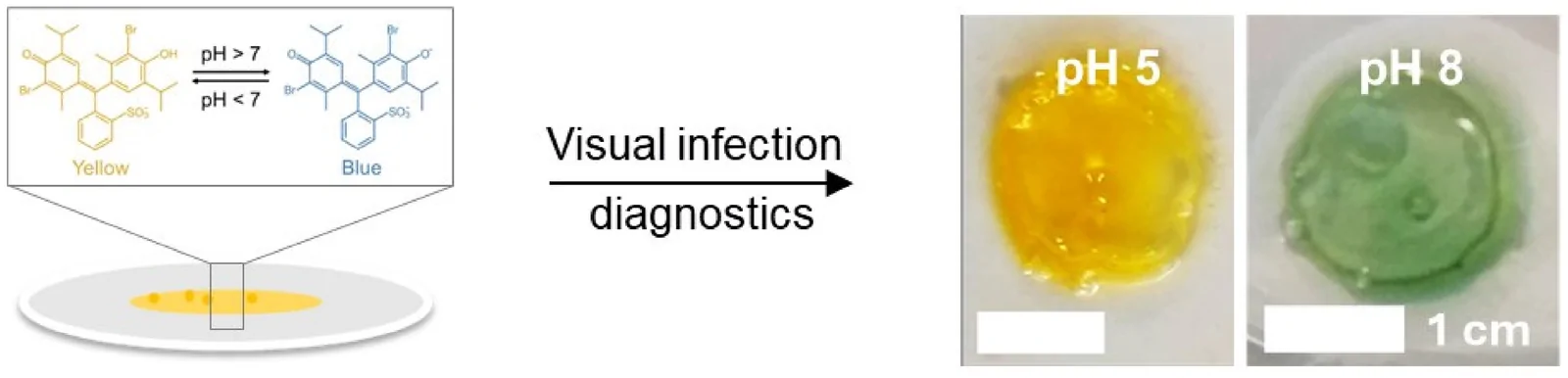
Drop-casting of a halochromic dye (bromothymol blue) onto a collagen substrate enables simple visual wound infection diagnostics through infection-associated pH elevation and pH-responsive colour change.

Effective infection management in chronic wounds relies not only on antimicrobial efficacy but also on the timely detection and monitoring of infection onset and progression. Conventional assessment methods are often subjective, dependent on late-stage clinical signs, and prone to overtreatment, contributing to unnecessary antimicrobial use. As a result, increasing attention has been directed toward wound dressings that integrate antimicrobial functionality with infection-indicating or -sensing capabilities, enabling earlier and more targeted interventions [202,203,204]. Recent advances in polymer science have enabled the development of smart wound dressings that respond to changes in the local wound microenvironment (such as pH shifts, enzymatic upregulation, oxidative stress, or bacterial metabolite production) to generate optical signals indicative of infection status (Figure 6).

These approaches include colorimetric, fluorescent, or opacity-based readouts that can be assessed directly by patients or caregivers without specialised instrumentation. By coupling infection detection with antimicrobial activation or guidance for intervention, such systems have the potential to support more rational treatment decisions, reduce empirical antibiotic use, and improve clinical outcomes. Importantly, stimulus-responsive antimicrobial platforms, including photodynamic and sonodynamic systems discussed in earlier sections [130,132,205,206], are particularly suited for integration with infection-sensing materials. In these hybrid systems, visual infection cues [207] may guide the timing and intensity of antimicrobial activation, enabling adaptive treatment strategies tailored to the evolving wound environment. This convergence of sensing, decision support, and responsive antimicrobial therapy represents a promising route toward intelligent wound-management technologies capable of improving infection control while reducing reliance on conventional antibiotics.

### 3.3. Medical Textiles for Infection Prevention

Over the past few years, significant progress has been made in developing antibiotic-free antimicrobial textiles to reduce pathogen transmission without relying on conventional biocides. A major focus has been on metal-free functional coatings, including plant-derived polyphenols [208], chitosan [209], graphene oxide [210], and quaternary ammonium compounds [211], which provide broad-spectrum antimicrobial activity through membrane disruption, charge-based interactions, and oxidative stress. Electrospun nanofibre-based layers incorporating biodegradable polymers and photocatalytic nanoparticles [212] have also gained traction, offering high filtration efficiency and rapid antibiotic-free bacterial inactivation. These materials are lightweight, breathable, and compatible with scalable manufacturing, making them highly suitable for medical and consumer face masks.

Another major direction involves stimulus-responsive and photoreactive systems, such as face masks coated with metal-free photocatalysts (e.g., carbon nitride, graphene-based materials) that generate reactive oxygen species under visible light, enabling self-sterilisation and prolonged reuse [213,214]. Similarly, triboelectric and plasma-treated fibres create surfaces that physically disrupt microbes or prevent adhesion without chemical leaching. Biological approaches, including antimicrobial peptides, enzymes, and phage-derived lysins immobilised on mask fibres, have shown strong activity against *S. aureus*, *E. coli*, and viral surrogates while maintaining breathability and safety [215]. Yim et al. performed a longitudinal study and examined the antimicrobial performance of PHMB-treated healthcare uniforms for a prolonged period of time and over repetitive laundry cycles in a hospital environment. The results from the study indicated the maintenance of over 99% of antimicrobial activity for up to 5 months, with no cases of AMR reported, indicating their potential use in hospital uniforms [216].

Research has also focused on the development of antibiotic-free antimicrobial surgical curtains to reduce risks of perioperative contamination and surgical-site infections in hospitals. Together with broad-spectrum antimicrobial efficacy, surgical curtains should also present suppressed biocide leaching, liquid and linting resistance, durability, and laundering compliance [217]. A major direction involves the design of metal-free functional coatings, including those made of chitosan, tannic acid, plant-derived polyphenols, and quaternary ammonium compounds, which provide broad-spectrum antibacterial activity through membrane disruption, charge-based interactions, and protein precipitation. Electrospun meshes incorporating natural polymers, graphene oxide, or photocatalytic agents have also been integrated into curtain fabrics to enhance filtration, reduce microbial adhesion, and inactivate bacteria on contact.

Self-sterilising and stimuli-responsive curtains incorporating photocatalytic materials such as carbon nitride, doped metal oxides, or graphene-based catalysts can generate ROS under ambient or surgical lighting, enabling continuous antimicrobial activity without chemical leaching [218,219]. Plasma-treated fibres, triboelectric surfaces, and nanotopographical textures have also been explored to physically disrupt bacterial membranes or prevent adhesion, offering long-lasting antimicrobial performance even after repeated sterilisation cycles. Emerging biological approaches such as antimicrobial peptides, enzyme-functionalised fibres, and phage-derived lysins provide targeted, non-antibiotic killing while supporting textile integrity and biocompatibility.

## 4. Challenges and Limitations

Despite the design principles outlined in Section 2.8, several key challenges continue to limit the industrial uptake and clinical translation of antibiotic-free antimicrobial approaches, including application-specific performance requirements, scalability, and regulatory considerations. Although cationic polymers, metal-oxide nanostructures, bacteriophages, and photodynamic therapies offer promising antibiotic-free antimicrobial solutions, microbial adaptation, evolutionary responses, and resistance development cannot be completely excluded, for example through membrane remodelling and altered surface charge, mirroring adaptive processes previously observed in response to conventional antibiotics. Biofilms remain particularly problematic, given that their dense extracellular matrix restricts nanoparticle (NP) penetration, quenches reactive oxygen species (ROS), and promotes adsorption of host-derived proteins that can mask contact-killing surfaces, thereby reducing antimicrobial efficacy. Long-term studies on microbial adaptation are therefore essential to assess the durability of antimicrobial performance, evaluate the potential emergence of reistance, and better understand broader ecological implications, including horizontal gene transfer.

Next, developing antimicrobial materials that balance potency, durability, and biocompatibility remains a major challenge for antibiotic-free antimicrobial approaches. Many promising agents, such as silver-based coatings, cationic polymers, and ROS-generating surfaces, exhibit narrow therapeutic windows, with antimicrobial activity often associated with risks of cytotoxicity, inflammation, or impaired tissue integration. In physiological environments, these materials may rapidly lose activity if the release kinetics of active factors are not controlled, limiting long-term performance on implants, catheters, and packaging surfaces. Manufacturing considerations further restrict scalability: plasma activation, photo-grafting, NP immobilisation, and multilayer coatings have yet to be standardised across industrial workflows, particularly for medical devices and stringent regulatory compliance. These engineering and safety constraints underscore the gap between laboratory-scale efficacy and the feasibility of producing durable, safe, and cost-effective functional materials at a commercial scale.

Finally, even when antibiotic-free technologies demonstrate strong antimicrobial potential, their clinical translation faces regulatory barriers as the translational evidence is currently sparse. The integrated antimicrobial modalities outlined in Section 3 sit at the interface of existing regulatory categories and thus require careful regulatory planning and industrial-scale manufacturing trials to demonstrate their commercialisation potential. Since regulatory considerations vary across territories, it is imperative to address potential translation barriers early in the design process. Standardised testing frameworks must also be optimised as conventional Minimum Inhibitory Concentration (MIC) and MBC assays are poorly suited for contact-killing surfaces, anti-adhesive coatings, or stimuli-responsive materials, resulting in inconsistent reporting across studies. Clinically, demonstrating superiority over antibiotics requires robust trials that address cost-effectiveness, day-to-day implementation, long-term safety, and clinically meaningful outcomes, criteria that few technologies have yet to meet. These systemic barriers highlight that technical innovation should be accompanied by regulatory compliance, validated testing standards, and sustainable economic models that support technology adoption for the benefit of patients, manufacturers, and health systems.

## 5. Conclusions and Future Directions

Building on the design strategies and current limitations discussed above, future research should focus on smarter, adaptive, and stimulus-responsive systems that activate antibiotic-free antimicrobial effects only in the presence of infection-specific cues. The next generation of antibiotic-free antimicrobial materials must evolve beyond passive bacterial killing towards multifunctional platforms capable of dynamically responding to the host–pathogen environment. Advances in polymer chemistry and nanotechnology are enabling coatings that switch between dormant and active states, improving selectivity and reducing unintended host damage. Bioinspired materials, including peptide-mimetic polymers, offer additional routes towards effective antimicrobial activity. These innovations aim to overcome major limitations of current materials, particularly their reduced efficacy in physiological environments and limited long-term durability on medical devices.

**Figure 7:**
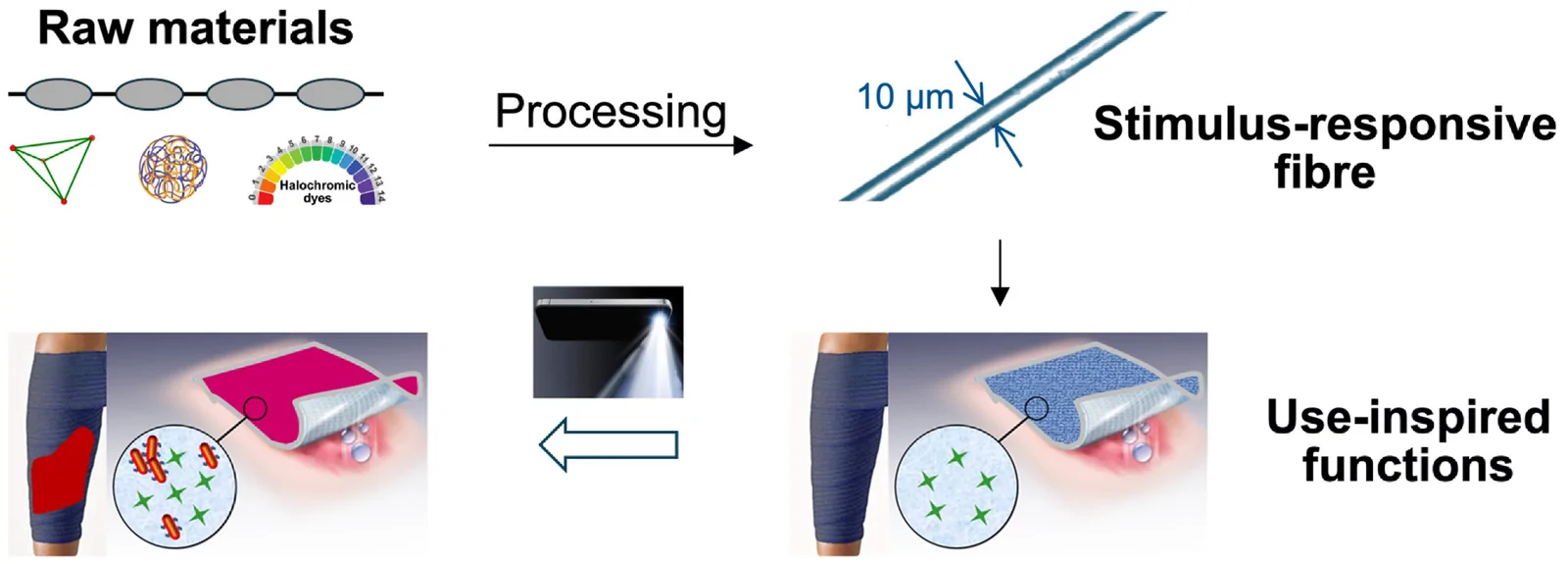
Innovations shaping the future of antibiotic-free materials for infection management and tissue repair. Textile fibres provide a versatile platform for integrating physical, chemical and biological mechanisms to enable infection detection, antimicrobial activation and tissue regeneration.

A major future direction for antibiotic-free antimicrobial materials is likely to involve the development of multi-modal platforms that combine physical, chemical, and biological mechanisms for prompt infection detection and user-driven antimicrobial activation. Among these, fibre-based systems are particularly attractive because they can integrate sensing, antimicrobial activity, and regenerative functions within a single platform (Figure 7). These innovations, along with advances in cationic chemistry, MOFs, photodynamic materials, and phage-derived enzymes, generate multifaceted antimicrobial pressures that are more difficult for microbes to circumvent. These hybrid systems also align with the growing interest in bio-integrated materials, where antimicrobial activity is paired with tissue compatibility, controlled degradation, and immune-modulating functions. Embedding sensing or diagnostic elements into antimicrobial surfaces, such as optical reporters or electrochemical biosensors, can enable real-time detection of colonisation or biofilm formation, transforming how implants, catheters, and wound dressings are monitored and managed in clinical practice.

Rather than simply eliminating microbes, these materials should simultaneously prevent protein adsorption, inhibit biofilm establishment, promote host cell integration, and accelerate healing. Such synergistic approaches are likely to outperform single-mechanism systems in complex clinical environments. Mechanisms of mechanical rupture, ROS generation, or multi-modal killing are inherently less vulnerable to classical resistance pathways, offering a potentially more sustainable long-term strategy.

Achieving this goal will require standardised preclinical testing, scalable manufacturing routes, regulatory approval pathways, and robust clinical evidence demonstrating long-term safety and efficacy. As materials become more selective, durable, and clinically validated, they could reshape infection prevention in orthopaedics and chronic wound management while supporting broader infection-control strategies across healthcare settings. Ultimately, these approaches have the potential to complement antibiotics, preserve their efficacy, and strengthen global resilience against antimicrobial resistance.

## Figures and Tables

**Table 1:** Types of surface modifications for antimicrobial activity and their associated biostatic or biocidal effects.

Surface Modification Method	Examples	Effect
Chemical grafting	Introduction of quaternary ammonium compounds (QACs), guanidines/biguanides, halogen-releasing compounds	Biocidal (contact-active or release-mediated) or biostatic
Surface coating or layer-by-layer (LbL)	Cationic polymer coatings (polyethylenimines, polyguanidines), LbL films incorporating antimicrobial compounds	Biocidal or biostatic
NP incorporation on polymer surfaces	Surface coating with metal nanoparticles	Metallic ion release and ROS production—biocidal
Plasma treatment + functionalisation	Introduction of reactive groups (–OH, –COOH, –NH_2_) onto polymer surfaces	Enhanced surface reactivity for subsequent biocidal functionalisation—indirect biocidal effect
Photosensitizer (PS) loading	Electrospinning of PS-loaded polymer solutions	Biocidal (light-activated)
Micro-/nanopatterning	Laser-textured surfaces that mechanically disrupt or deter bacterial adhesion	Biocidal (via mechanical action) or biostatic (via anti-adhesive action)
Hydrophilic surface modification	Surface PEGylation to prevent microbial adhesion	Biostatic (anti-adhesive/repulsive effect)
Antimicrobial drug immobilisation	Antibiotic-loaded polymer brushes, controlled-release coatings	Sustained antimicrobial release—biocidal

**Table 2:** Comparison of the structural and antimicrobial characteristics of metal-organic frameworks (MOFs), antimicrobial peptides (AMPs) and antimicrobial proteins.

Performance Parameter	MOFs	AMPs	Antimicrobial Proteins
Primary antimicrobial mechanism	Metal-ion release, ROS generation, membrane disruption, photocatalytic killing, drug delivery	Membrane disruption, intracellular targeting, antibiofilm activity, immune modulation	Enzymatic cell-wall degradation, immune-mediated killing, pathogen-specific targeting
Spectrum of activity	Broad; bacteria, fungi, some viruses	Broad; effective against many MDR pathogens	Usually narrower and more specific
Risk of resistance	Low due to multi-modal antimicrobial mechanisms	Generally low but not excluded	Lower than many antibiotics; immune evasion possible
Structural tunability	Excellent; metal, linker, pore and surface engineering	High through sequence engineering	High through protein engineering
Drug-delivery capability	Excellent carrier with controlled release	Limited intrinsic carrying ability	Usually requires delivery systems
Stability	Tunable; stability depends strongly on framework composition and microenvironment	Prone to protease degradation and short half-life	Susceptible to denaturation and proteolysis
Biocompatibility	Variable; toxicity depends on composition	Generally good but haemolysis/toxicity can occur	Usually high but immunogenicity possible
Manufacturing	Potentially scalable chemical synthesis; standardisation challenging	Peptide synthesis can be expensive	Recombinant production and purification costly
Clinical translation	Mostly preclinical	Some in trials/limited use	Some advanced therapeutic proteins available
Applicability	Coatings, implants, wound dressings, delivery systems	Topical/systemic anti-infectives, biofilms	Precision therapeutics and adjunctive therapies

## Data Availability

No new data were created or analyzed in this study. Data sharing is not applicable to this article.

## Supplementary Materials

The following supporting information can be downloaded at: https://www.mdpi.com/article/10.3390/pharmaceutics18091136/s1, Table S1: Representative examples of recently reported antimicrobial metal-organic frameworks (MOFs) and their antimicrobial activity; and Table S2: Representative antimicrobial peptides and proteins reported in recent literature.
