# Supplementary material for: Antibiotic-Free Strategies for Managing Antimicrobial-Resistant Infections
Source: Pharmaceutics. 2026 Sep 10;18(9):1136. doi: 10.3390/pharmaceutics18091136 (PMC13610601; doi:10.3390/pharmaceutics18091136)
Supplement: Supplementary file 1 [file pharmaceutics-18-01136-s001.zip › pharmaceutics-4457874-supplementary.pdf]

Supplementary tables for section 2.4

Table S1. Recently Developed Antimicrobial MOFs

MOF material	MIC	Target microorganism(s)	Reference
Ag-MOF (silver-based metal-organic framework)	10 μg mL^{-1}	Multidrug-resistant Klebsiella pneumoniae	Hassan et al., Nanoscale Advances, 2024
Cu-BTC MOF	Complete inhibition at ~1500 μg dose	Escherichia coli, Lactobacillus spp.	Elmehrath et al., RSC Advances, 2024
Cu-GA MOF	Complete inhibition at ~2000 μg dose	Escherichia coli, Lactobacillus spp.	Elmehrath et al., RSC Advances, 2024
Zn-based ZIF-type MOFs	Typically tens to hundreds of μg mL^{-1}	Staphylococcus aureus, E. coli	Yang et al., Nano Research, 2022
Cu-based antibacterial MOFs	Composition-dependent	Gram-positive and Gram-negative pathogens	Yang et al., Nano Research, 2022

Table S2. Antimicrobial Biomolecules Reported During the Last Decade

Biomolecule	Type	MIC	Target microorganism(s)	Reference
GW18	Synthetic antimicrobial peptide	Active against S. aureus and MRSA (strain-dependent)	S. aureus, MRSA	Yuan et al., Frontiers in Microbiology, 2022
mKLK	Engineered antimicrobial peptide	Potent activity against planktonic and biofilm strains	S. aureus	Akhash et al., Scientific Reports, 2024
Pexiganan	Host-defense peptide analogue	Low μg mL^{-1} range	S. aureus	Rodriguez-Rojas et al., Frontiers in Immunology, 2020
ClyC	Chimeric phage lysin	1-12 μg mL^{-1}	S. aureus, MRSA	Li et al., Antibiotics, 2021
Exebacase (CF-301)	Recombinant lysin	Low μg mL^{-1} range	S. aureus including resistant isolates	McCarthy M.W., Drugs, 2022

References:

1. Hassan, P. B., Mohammed Ameen, S. S., Mohammed, L., Muhammed Ameen, S. M., & Omer, K. M. (2024). Enhanced antibacterial activity of a novel silver-based metal organic framework towards multidrug-resistant *Klebsiella pneumonia*. *Nanoscale advances*, *6*(15), 3801–3808. https://doi.org/10.1039/d4na00037d
2. Elmehrath, S., Ahsan, K., Munawar, N., Alzamly, A., Nguyen, H. L., & Greish, Y. (2024). Antibacterial efficacy of copper-based metal-organic frameworks against *Escherichia coli* and *Lactobacillus*. *RSC advances*, *14*(22), 15821–15831. https://doi.org/10.1039/d4ra01241k
3. Yang, M., Zhang, J., Wei, Y., Zhang, J., & Tao, C. (2022). Recent advances in metal-organic framework-based materials for anti-staphylococcus aureus infection. *Nano research*, *15*(7), 6220–6242. https://doi.org/10.1007/s12274-022-4302-x
4. Yuan, B., Lu, X., Yang, M., He, Q., Cha, Z., Fang, Y., Yang, Y., Xu, L., Yan, J., Lai, R., Wang, A., Yu, X., & Duan, Z. (2022). A designed antimicrobial peptide with potential ability against methicillin resistant *Staphylococcus aureus*. *Frontiers in microbiology*, *13*, 1029366. https://doi.org/10.3389/fmicb.2022.1029366
5. Akhash, N., Farajzadeh Sheikh, A. & Farshadzadeh, Z. Design of a novel analogue peptide with potent antibiofilm activities against *Staphylococcus aureus* based upon a sapecin B-derived peptide. *Sci Rep* **14**, 2256 (2024). https://doi.org/10.1038/s41598-024-52721-0
6. Rodríguez-Rojas, A., Nath, A., El Shazely, B., Santi, G., Kim, J. J., Weise, C., Kuropka, B., & Rolff, J. (2020). Antimicrobial Peptide Induced-Stress Renders *Staphylococcus aureus* Susceptible to Toxic Nucleoside Analogs. *Frontiers in immunology*, *11*, 1686. https://doi.org/10.3389/fimmu.2020.01686
7. Li, X., Wang, S., Nyaruaba, R., Liu, H., Yang, H., & Wei, H. (2021). A Highly Active Chimeric Lysin with a Calcium-Enhanced Bactericidal Activity against *Staphylococcus aureus* In Vitro and In Vivo. *Antibiotics (Basel, Switzerland)*, *10*(4), 461. https://doi.org/10.3390/antibiotics10040461
8. McCarthy M. W. (2022). Exebacase: A Novel Approach to the Treatment of Staphylococcal Infections. *Drugs in R&D*, *22*(2), 113–117. https://doi.org/10.1007/s40268-022-00383-6
